# Type 1 diabetes mellitus and its glycemic control affect sleep

**DOI:** 10.1186/1758-5996-7-S1-A49

**Published:** 2015-11-11

**Authors:** Mark Thomaz Ugliara Barone, Anna Beatriz Ayumi-Sato, Etienne Yoko Mitsuuchi, Daniela Wey, Fabíola Schorr, Denise R Franco, Mario Kehdi Carra, Geraldo Lorenzi-Filho, Viviana Giampaoli, Luiz Menna-Barreto

**Affiliations:** 1 GMDRB/EACH/USP & ADJ, São Paulo, Brazil

## Background

In the current and past decades, associations between sleep impairment and glucose control in individuals with diabetes have been unveiled. Sleep disorders and curtailment were shown to deteriorate glycemic control, while glycemic extremes impact sleep quality and melatonin secretion, forming a vicious circle.

## Objectives

Our aim with the present study was to identify associations between glycemic control and parameters of the sleep-wake cycle, with emphasis on the sleep quality, of adults with type 1 diabetes mellitus (T1D), a population poorly studied in the sleep field.

## Materials and methods

Eighteen non-obese adults with T1D (8 males, age=26.3±5.1), without chronic complications were studied. The following instruments were used during ten consecutive days: sleep diaries (with Visual Analogue Scales for rating sleep quality), actimeters, and a home fingertip glucometer (6.41±1.5 tests a day). The analysis was made using fitted inflated beta regression models with GAMLSS for the response variable sleep quality rate (SQR). Subject-level covariables in this dataset included: number of hyperglycemia and hypoglycemia episodes in the previous day, number of awakenings during the night, morning awakening type (spontaneous or not), sleep rate (total sleep time in min divided by 1440), and the corresponding interactions.

## Results

The SQR was negatively associated with the sleep rate (p<0.001), and positively associated with spontaneous awakening (p=0.042). The relationships between the other variables with SQR were the following: the SQR rises with the increase of both, hypoglycemic events and sleep rate (p=0.002). On the other hand, when the hypoglycemic or hyperglycemic events are associated with non-spontaneous awakening, the SQR decreases (p=0.02 for both glycemic conditions). However, the SQR increases when both the number of hyperglycemia and the number of night awakenings increase (p=0.01).

## Conclusion

The present study contributes with a specific understanding that T1D, especially through its glycemic excursions, impacts the sleep-wake cycle in different ways, with emphasis on the sleep quality. Thus, the previously proposed vicious circle involving sleep and diabetes is reinforced. Considering that, health care professionals should be encouraged to pay attention to sleep patterns and complaints of patients, in order to identify and address hidden poor glycemic control, and, this way, help to improve this population's sleep and life quality.

